# 

**Published:** 1887-06

**Authors:** 


### CONTENTS FOR JUNE, '87, VOL. II, NO. 12.

ORIGINAL COMMUNICATIONS.—A Practical Test in the Treatment of Pulmonary Phthisis by Gaseous Enemata, by C. A. Parker, M. D., Fort Worth ..................... 503

Methods in Electricity, by F. T. Paine, M. D., Comanche 506

SOCIETY NOTES.—Resolutions by Galveston Medical Club on the Death of Dr. W. S. Rogers .................... 508

Notes from the June Meeting of the American Medical Asssociation ........................................ 509

East Texas Medical Association........................ 510

Address of the President Texas State Medical Association in Interest of the International Medical Congress..... 511

Houston County Medical Association.................... 513

MEDICAL NEWS AND MISCELLANY.—Tulane University, Medical Department—Another Press Victimizer—International Congress on Inebriety—Dr. Stone not Dead—Yellow Fever Threatening Texas—A New District Medical Society to be Organized..........................514-515

CULLINGS FROM CONTEMPORARIES.—Ligature of Common Carotid Artery in an Abscess following Diphtheritic Scarlatina; Recovery.................................. 515

Successful Enucleation of Fibroid, after Laparotomy .... 510

CONTRIBUTED—Address of Dr. T. D. Wooten, President Board of Regents. Delivered beforc the Texas State Medical Association, by Invitation, on the Status of the State University ........................................ 519

EDITORIAL—To the Patrons of the JOURNAL—End of Volume II; Beginning of Volume III .................... 536

ADVERTISERS' NOTICES.................................. 537

